# Three New Ursane-Type Triterpenoids from the Stems of *Saprosma merrillii*

**DOI:** 10.3390/molecules181214496

**Published:** 2013-11-25

**Authors:** Dashuai Zhang, Wenhao Chen, Wenxing Chen, Xiaoping Song, Changri Han, Yan Wang, Guangying Chen

**Affiliations:** 1 Key Laboratory of Tropical Medicinal Plant Chemistry of Ministry of Education, College of Chemistry and Chemical Engineering, Hainan Normal University, Haikou 571158, Hainan, China; 2 Jiangsu Key Laboratory for Pharmacology and Safety Evaluation of Chinese Materia Medica, Nanjing University of Chinese Medicine, Nanjing 210046, Jiangsu, China

**Keywords:** *Saprosma merrillii*, bioassay-guided fractionation, triterpenoid, antineoplastic activity

## Abstract

Three new ursane-type triterpenoids, 3*α*,6*α*,30-trihydroxy-ursan-28-oic acid (**1**), 3*α*,30-dihydroxy-6-oxo-ursan-28-oic acid (**2**) and 3*α*,6*α*,7*α*,30-tetrahydroxy-ursan-28-oic acid (**3**), together with one known triterpenoid, betulinic acid (**4**), one known anthraquinone, 1,7-dihydroxy-2-methylanthraquinone (**5**), four known phenols, 1,3,5-trimethoxybenzene (**6**), *p*-hydroxybenzoic acid (**7**), syringic acid (**8**), isovanillin (**9**), two steroids, sitosterol (**10**) and daucosterol (**11**), were isolated from the ethanol extract of the stems of *S. merrillii*. Their structures were elucidated on the basis of physical and spectral techniques, besides comparison with literature data. Compounds **1**–**3** showed inhibitory activities against the A549, HEPG2, and B16F10 cell lines.

## 1. Introduction

The genus *Saprosma* belongs to the family Rubiaceous which has about 50 species found around the world, and has been used as a traditional medicine for the treatment of fever and lumbocrural pain [1,2,3,4,5,6]. Iridoid glycosides [1,2,7], sulfur-containing iridoid glycosides [1,7,8,9], anthraquinones [3,7,9], and alkaloids [4] have been reported from this genus, and some of these compounds showed good anti-inflammatory, antibacterial and antitumor effect [1,2,3,4].

**Figure 1:**
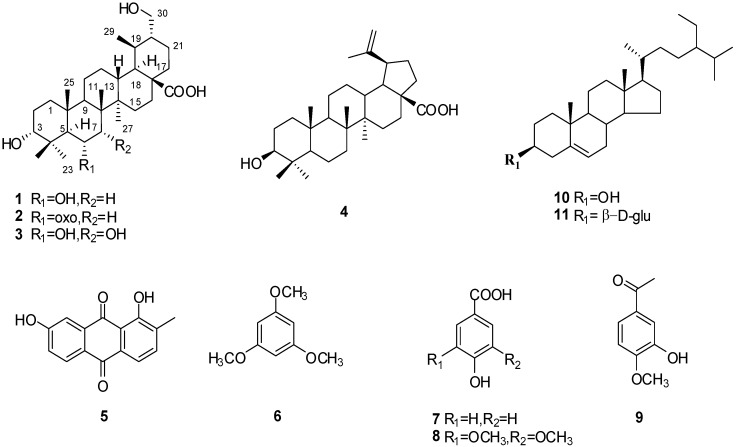
Structures of compounds **1–11**.

*S. merrillii *is an endemic plant in Hainan Island whose chemical constituents have not been investigated previously. As part of our continuing study on bioactive components from tropical medicinal plants from Hainan, the stems of *S. merrillii* were investigated. The EtOAc soluble fraction of the EtOH extract of *S. merrillii*, which showed cytotoxic activity against the A549 cell line, with an IC_50_ value of 65.66 μg/mL, was further purified by column chromatography to afford three new ursane-type triterpenoids **1**–**3** (Figure 1) and eight known compounds **4**–**11**. Here, we wish to report on the isolation and structural elucidation of these compounds. Compounds **1**–**3** were evaluated for their cytotoxicity against the A549, MDA-MB-231, HEPG2 and B16F10 tumor cell lines.

## 2. Results and Discussion

The air-dried stems of *S. merrillii* were extracted three times with 80% EtOH at room temperature. The EtOH extract of the plant was suspended in water and then partitioned successively with petroleum ether and EtOAc. Column chromatography (CC) of the EtOAc-soluble fraction yielded three new compounds **1**–**3** and eight known compounds **4**–**11**.

Compound **1** was isolated as a white amorphous solid. Its molecular formula was assigned to be C_30_H_50_O_5_ based on the HRESIMS (*m/z*: 513.3559 [M+Na]^+^, calcd. for C_30_H_49_O_5_Na, 513.3551) indicating six degrees of unsaturation. The IR spectrum showed absorption bands for hydroxyl (3,432 cm^–1^), carbonyl (1,704 cm^–1^) and C-O (1,040 cm^–1^) functions, respectively.

**Table 1:** NMR data for compounds **1**–**3** (a in MeOD, b in acetone-d_6_, *δ* in ppm, *J* in Hz).

NO.	1 ^a^		2 ^b^		3 ^b^	
	*δ*_C_	*δ*_H_	*δ*_C_	*δ*_H_	*δ*_C_	*δ*_H_
1	38.3 t	1.54 (1H, m, H-1β), 1.63 (1H, m, H-1α)	34.5 t	1.47 (1H, m, H-1β), 1.65 (1H, m, H-1α)	34.2 t	1.64 (1H, m, H-1β), 1.83 (1H, m, H-1α)
2	26.4 t	1.51 (1H, m, H-1α), 2.05 (1H, m, H-1β)	26.0 t	1.56 (1H, m, H-1α), 1.92 (1H, m, H-1β)	26.6 t	1.50 (1H, m, H-1α), 2.04 (1H, m, H-1β)
3	78.5 d	3.26 (1H, t, 2.4)	75.7 d	3.19 (1H, br s)	77.0 d	3.28 (1H, t, 2.6)
4	39.4 s		37.2 s		39.1 s	
5	50.2 d	1.31 (1H, br.s)	60.0 d	2.77 (1H, s)	48.7 d	1.41 (1H, br.s)
6	69.3 d	4.34 (1H, m)	213.7 s		73.9 d	4.11 (m)
7	42.9 t	1.50 (1H, m, H-1α), 1.63 (1H, m, H-1β)	52.8 t	1.80 (1H, m, H-1α), 2.57 (1H, d, 11.6 H-1β)	74.6 d	3.62 (1H, dd, 4.4, 4.8)
8	41.3 s		48.2 s		47.0 s	
9	49.8 d	1.63 (1H, m)	49.3 d	1.73 (1H, m)	49.2 d	1.70 (1H, m)
10	38.0 s		44.3 s		37.8 s	
11	22.1 t	1.43 (1H, m, H-1α), 1.61 (1H, m, H-1β)	22.1 t	1.41 (1H, m, H-1α), 1.66 (1H, m, H-1β)	21.7 t	1.39 (1H, m, H-1α), 1.64 (1H, m, H-1β)
12	33.2 t	1.37 (1H, m, H-1β) 2.22 (1H, dt, 12.6, 2.8 H-1α)	32.6 t	1.48 (1H, m, H-1β), 2.24 (1H, m, H-1α)	33.1 t	1.35 (1H, m, H-1β), 2.20 (1H, dt, 12.8, 3.6 H-1α)
13	38.8 d	2.41 (1H, dt, 12.2, 3.6)	39.1 d	2.37 (1H, m)	38.9 d	2.41 (1H, dt, 12.8, 3.6)
14	43.9 s		43.8 s		44.8 s	
15	31.0 t	1.17 (1H, m, H-1β), 1.56 (1H, m, H-1α)	30.4 t	1.02 (1H, m, H-1β), 1.48 (1H, m, H-1α)	36.6 t	1.16 (1H, m, H-1β), 1.34 (1H, m, H-1α)
16	28.6 t	1.34 (1H, m, H-1α), 1.66 (1H, m, H-1β)	27.6 t	1.43 (1H, m, H-1α), 1.69 (1H, m, H-1β)	28.5 t	1.28 (1H, m, H-1α), 1.66 (1H, m, H-1β)
17	57.9 s		57.0 s		57.0 s	
18	51.9 d	1.54 (1H, m)	51.0 d	2.08 (1H, t, 4.0)	51.6 d	1.40 (1H, m)
19	39.5 d	1.83 (1H, m)	38.8 d	1.86 (1H, m)	39.3 d	1.87 (1H, m)
20	44.6 d	2.31 (1H, tt, 10.8, 3.0)	44.2 d	2.29 (1H, m)	44.4 d	2.30 (1H, m)
21	24.7 t	1.31 (1H, m, H-1α), 1.51 (1H, m, H-1β),	24.4 t	1.39 (1H, m, H-1α), 1.55 (1H, m, H-1β)	24.4 t	1.32 (1H, m, H-1α), 1.54 (1H, m, H-1β)
22	36.8 t	1.30 (1H, m, H-1α), 1.37 (1H, m, H-1β)	37.7 t	1.34 (1H, m, H-1α), 1.37 (1H, m, H-1β)	37.9 t	1.32 (1H, m, H-1α), 1.35 (1H, m, H-1β)
23	28.9 q	0.98 (3H, s)	27.4 q	0.94 (3H, s)	28.7 q	0.98 (3H, s)
24	24.8 q	1.20 (3H, s)	22.6 q	1.19 (3H, s)	25.0 q	1.22 (3H, s)
25	15.4 q	0.99 (3H, s)	17.4 q	0.86 (3H, s)	15.6 q	1.06 (3H, s)
26	17.4 q	1.28 (3H, s)	16.5 q	0.93 (3H, s)	11.0 q	1.21 (3H, s)
27	18.0 q	1.23 (3H, s)	15.1 q	1.14 (3H, s)	17.8 q	1.22 (3H, s)
28	180.2 s		177.7 s		177.8 s	
29	18.8 q	0.96 (3H, d, 6.8)	18.8 q	0.93 (3H, d, 7.0)	18.8 q	0.94 (3H, d, 6.8)
30	64.2 t	3.33 (1H, m, H-1β), 3.75 (1H, dd, 10.6, 4.4, H-1α)	63.7 t	3.37 (1H, dd, 10.0, 8.4, H-1β), 3.76 (1H, dd, 10.0, 4.6, H-1α)	63.8 t	3.34 (1H, m, H-1α), 3.76 (1H,10.2, 4.6, H-1β)

The ^1^H-NMR spectrum of **1** showed the following signals: five tertiary methyl groups at *δ*_H_ 1.28, 1.23, 1.20, 0.99 and 0.98 (each 3H, s), a secondary methyl at *δ*_H_ 0.96 (3H, d, *J *= 6.8Hz, H-29), one oxygenated methylene protons *δ*_H_ 3.75 (1H, dd, *J *= 10.6, 4.4 Hz, H-30b), and 3.33 (1H, m, H-30a), two oxygenated methine protons *δ*_H_ 4.34 (1H, m, H-6), 3.26 (1H, t, *J *= 2.4 Hz, H-3) and a series of overlapped signals suggesting an ursane-type triterpenoid. The ^13^C-NMR spectrum revealed 30 carbon signals, which were further classified by DEPT and HSQC experiments as six methyls, 10 methylenes (one oxygenated), eight methines (two oxygenated), six quaternary carbons (including one carboxyl group) (Table 1). The aforementioned data implied that **1** was an ursane-28-oic-acid with three hydroxyls [10,11,12,13,14,15].

**Figure 2:**
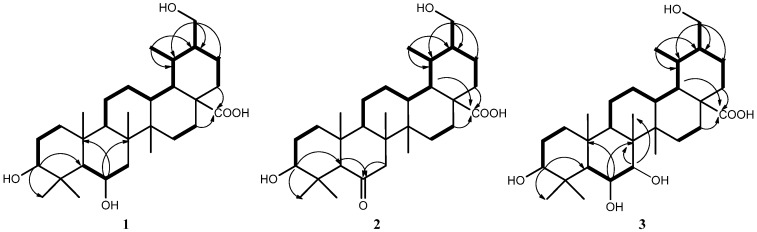
Key ^1^H-^1^H COSY (bold bonds) and HMBC correlations (arrows) of **1–3**.

In the HMBC spectrum, the oxygenated methylene protons *δ*_H_ 3.75, 3.33 (H_2_-30) showed correlations to C-19 (*δ*_C_ 39.5), C-20 (*δ*_C _44.6) and C-21 (*δ*_C_ 24.7), indicating that one hydroxyl was linked at C-30. Moreover, two oxygenated methine protons *δ*_H_ 4.34 (H-6) and *δ*_H_ 3.26 (H-3), which were deduced by the HMBC correlations from the proton *δ*_H_ 4.34 to C-8 (*δ*_C_ 41.3), C-10 (*δ*_C_ 38.0) and from *δ*_H_ 3.26 to C-5 (*δ*_C_ 50.2), C-23 (*δ*_C_ 28.9) and C-24 (*δ*_C_ 24.8). These observations, together with the HMBC correlations between *δ*_H_ 1.34, 1.66 (H_2_-16) and 1.30, 1.37 (H_2_-22) and the carboxyl group *δ*_C_ 180.2 (C-28), were used to establish the molecular framework of **1** (Figure 2).

**Figure 3:**
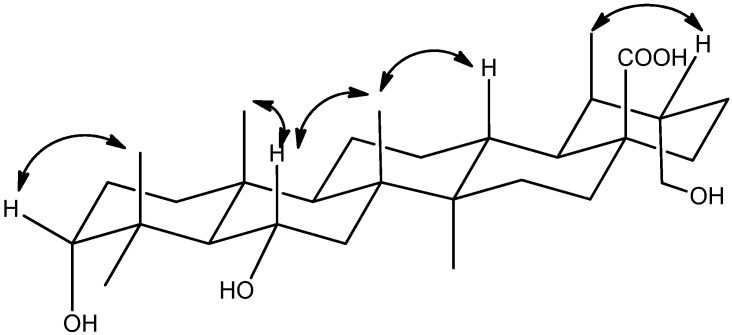
Key NOESY correlations of **1**.

The relative configuration of **1** was established by analysis of the key correlations displayed in the NOESY spectrum (Figure 3). The key correlation from *δ*_H_ 3.26 (H-3) to 1.20 (H_3_-24), proves hydrogen of C-3 is *β*-orientation. *δ*_H_ 4.34 (H-6) showed correlations to *δ*_H_ 0.99 (H_3_-25) and 1.28 (H_3_-26), proves hydrogen of C-6 is *β*-orientation. On the basis of the above discussion, the relative configuration of **1** was established as shown (Figure 1). Thus, compound **1** was determined to be 3*α*,6*α*,30-trihydroxyursan-28-oic acid.

**Figure 4:**
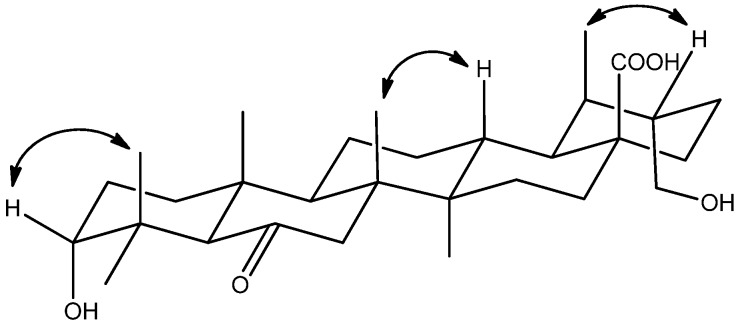
Key NOESY correlations of **2**.

Compound **2** was isolated as a white amorphous solid. Its molecular formula was assigned to be C_30_H_48_O_5_ based on the HRESIMS, which showed a unit of two hydrogen atoms less than that of compound **1**. Its NMR spectra (Table 1 and Experimental Section) were similar to those of **1**, except for one oxygenated methine *δ*_H_ 4.34 (1H, m, H-6), *δ*_C_ 69.3 (C-6) to the ketone carbon at *δ*_C_ 213.7 (C-6) in **2**. This was supported by the HMBC correlations between *δ*_H_ 2.77 (H-5), 1.80 and 2.57 (H_2_-7) and the carbonyl group (Figure 2). The relative configuration of compound **2** was obtained from the NOESY spectrum (Figure 4), the cross-peak between *δ*_H_ 3.19 (H-3) and 1.19 (H-24) indicated that the configuration of **2** was the same as that shown above for **1**, in relevant parts of the molecule. Finally, the structure of **2** was determined to be 3*α*,30-dihydroxy-6-oxo-ursan-28-oic acid.

**Figure 5:**
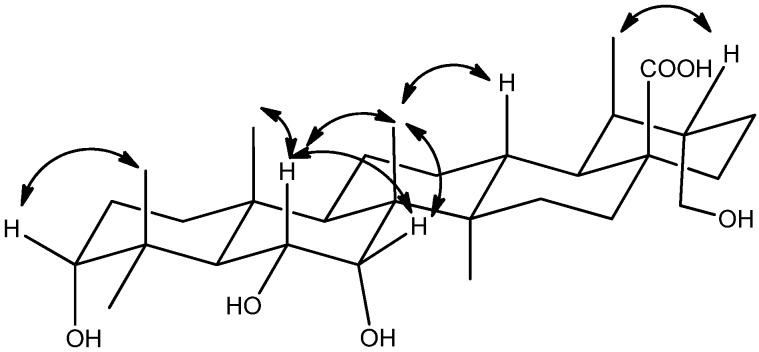
Key NOESY correlations of compound **3**.

Compound **3** was isolated as a white amorphous solid. Its molecular formula was assigned to be C_30_H_50_O_6 _based on the HRESIMS, which showed a unit of one oxygen atom more than that of compound **1**. Its NMR spectra (Table 1 and Experimental Section) were similar to those of **1**, except for one more oxygenated methine *δ*_H _3.62 (1H, t, *J *= 4.8 Hz, H-7) that showed correlation to *δ*_H_ 4.11 (H-6) in the ^1^H-^1^H COSY spectrum fixed its position. In the NOESY spectrum (Figure 5), the key correlations from *δ*_H_ 4.11 (H-6) to 1.06 (H_3_-25), *δ*_H _3.62 (H-7) to 1.21 (H_3_-26), proves hydrogens of C-6 and C-7 are both *β*-orientation. Thus, the structure of compound **3** was elucidated as 3*α*,6*α*,7*α*,30-tetrahydroxy-ursan-28-oic acid.

Compounds **1**–**3** were evaluated for their anti-proliferative activities against the human lung cancer A549, human breast cancer MDA-MB-231, human hepatocellular carcinoma HEPG2 and mouse melanoma B16F10 cancer cell lines using the 3-(4,5-dimethylthiazol-2-yl)-2,5-diphenyltetrazolium bromide (MTT) assay method *in vitro* [16]. Compound **1** exhibited weak cytotoxicity against the B16F10 cell line, with an IC_50_ value of 72.72 μM, while **2** inhibited the proliferation of the A549 cell line with an IC_50_ value of 24.66 μM (Table 2).

**Table 2:** Cytotoxic activities of **1**–**3** against four cancer cell lines.

Compounds	IC_50_ (μM)			
	A549	MDA-MB-231	HEPG2	B16F10
**1**	N	N	N	72.72
**2**	24.66	N	127.80	N
**3**	129.22	N	N	N

N: means inactive with IC_50_ values above 150 μM.

## 3. Experimental

### 3.1. General

Optical rotations were measured with PolAAr 3005 polarimeter (Optical Activity Limited, Cambridgeshire, UK). IR spectra were recorded on a Thermo Nicolet 6700 (using KBr disks) spectrophotometer (Thermo Scientific, Madison, WI, USA). NMR spectra were acquired on a Bruker AV (400 MHz for 1H-NMR, ppm relative to TMS) spectrometer (Bruker, Hesse-Darmstadt, Germany). ESIMS spectra were recorded on an Agilent 1200 series HPLC (Agilent Technologies, Waldbroon, Germany) interfaced to an Bruker Esquire 6000 Ion Trap mass spectrometer equipped with an electrospray ionization source, and HRESIMS spectra were made on the Bruker Daltonics Apex-Ultra 7.0 T (Bruker Corporation, Billerica, MA, USA), respectively. Column chromatography of silica gel (200–300 mesh), all solvents for column chromatography were of analytical grade (Xilong Chemical Reagents Company, Ltd., Guangdong, China). Spots of compounds on TLC were visualized by spraying using 10% H_2_SO_4_ in EtOH (v/v) followed by heating.

### 3.2. Plant Material

*S. merrillii* was collected from Sanya, Hainan Province, China, in August 2009 and was identified by Professor Qiongxin Zhong of School of Life Science, Hainan Normal University. A voucher specimen (No. 200908QDRMS) was deposited at the Department of Key Laboratory of Tropical Medicinal Plant Chemistry of Ministry of Education, Hainan Normal University.

### 3.3. Extraction and Isolation

The stems of *S. merrillii* was air-dried (9.8 kg) and extracted three times with 80% EtOH (60 L × 3 d each) at room temperature. The EtOH extract (380 g) was suspended in water and then partitioned successively with petroleum ether and EtOAc. The EtOAc-soluble portion (60 g) was subject to Si gel CC (200–300 mesh), eluting with a gradient of mixtures of petroleum ether/EtOAc/MeOH (from 1:0:0 to 0:0:1) to give 10 major fractions (Frl–Fr10) according to TLC analysis. Frl (1.1 g) was recrystallized from EtOAc to afford **10** (0.5 g). Fr2 (0.2 g) was chromatographed on a Si gel column eluted with petroleum ether/EtOAc (from 1:0 to 1:1) to afford **4** (1.5 mg). Fr3 (0.4 g) was chromatographed on a Si gel column eluted with petroleum ether/EtOAc (from 1:0 to 1:2) to afford **5** (2.5 mg) and **6** (2.0 mg). Fr4 (0.5 g) was chromatographed on a silica gel column eluted with petroleum ether/EtOAc (from 1:0 to 1:2) to afford eight major subfractions (Fr4a–Fr4h). Fr4a (0.3 g) was further purified by Si gel column chromatography (petroleum ether/EtOAc, from 1:0 to 1:2) to obtain **7** (3.0 mg). Fr5 (0.6 g) was chromatographed on Si gel column eluted with petroleum ether/acetone (from 2:1 to 0:1) to afford **8** (3.5 mg). Fr6 (0.5 g) was chromatographed on Si gel column eluted with petroleum ether/CHC1_3_/EtOAc (4:2:1) to afford six major subfractions, Fr6a–Fr6f. Fr6b (0.2 g) was further purified by Si gel (petroleum ether/CHC1_3_/EtOAc, 2:4:1) to obtain **9** (2.0 mg). Fr7 (1.2 g) was chromatographed on Si gel column eluted with petroleum ether/EtOAc (from 1:0 to 0:1), recrystallized from MeOH to afford **1** (53 mg). Fr8 (1.2 g) was chromatographed on Si gel column eluted with petroleum ether/EtOAc (from 1:0 to 0:1), recrystallized from acetone to afford **2** (15 mg). Fr9 (1.2 g) was chromatographed on Si gel column eluted with petroleum ether/EtOAc (from 1:0 to 0:1), recrystallized from acetone to afford **3** (10.0 mg). Fr10 (5.0 g) was recrystallized from MeOH to afford **11** (1.1 g).

### 3.4. Characterization of Compounds **1**–**3**

Compound **1**: white amorphous solid; 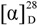 −108.33 (*c* 0.0024, MeOH); IR (KBr) υ_max_ 3432, 2949, 2873, 1704, 1641, 1459, 1383, 1278, 1181, 1040, 990 cm^–1^; ^1^H- and ^13^C-NMR data see Table 1; ESIMS *m/z* 489.4 [M−H]^−^, 513.3 [M+Na]^+^; HRESIMS *m/z*: 513.3559 [M+Na]^+^ (calcd for C_30_H_49_O_5_Na, 513.3551).

Compound **2**: white amorphous solid; 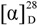 −20.73 (*c* 0.0019, MeOH); IR (KBr) υ_max_ 3440, 2948, 2873, 1704, 1636, 1458, 1387, 1174, 1070, 1032, 986, 928 cm^–1^; ^1^H- and ^13^C-NMR data see Table 1; ESIMS *m/z*: 487.6 [M−H]^−^, 511.5 [M+Na]^+^; HRESIMS *m/z*: 511.3389 [M+Na]^+^ (calcd for C_30_H_48_O_5_Na, 511.3394).

Compound **3**: white amorphous solid; 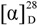 –37.84 (*c* 0.0037, MeOH); IR (KBr) υ_max_ 3442, 2951, 2875, 1704, 1647, 1458, 1384, 1229, 1176, 1110, 1035, 920 cm^–1^; ^1^H- and ^13^C-NMR data see Table 1; ESIMS *m/z*: 505.7 [M−H]^−^, 529.5 [M+Na]^+^; HRESIMS *m/z*: 529.3501 [M+Na]^+^ (calcd for C_30_H_50_O_6_Na, 529.3499).

### 3.5. Cytotoxicity Assays

Test compounds **1**–**3** were dissolved in DMSO (final concentration, 0.1%). The cytotoxicity of compounds **1**–**3** against A549, MDA-MB-231, HEPG2 and B16F10 was determined by standard MTS assay [16]. Cells dispersed evenly in medium and were seeded in a 96-well plate with a density of 1 × 10^4^ cells/well. Next day, cells were treated with various concentrations of samples (0–100 μM) for 48 h or treated with indicated concentrations for 48 h with six replicates of each treatment. After incubation, each well was added 20 μL of MTS [3-(4,5-dimethylthiazol-2-yl)-5-(3-car-boxymethoxy-phenyl)-2-(4-sulfophenyl)-2*H*-tetrazolium] reagent and incubated for 3 h. Cell viability was determined by measuring the optical density at 490 nm using a Biotek microplate reader (Biotek, Winooski, VT, USA). Untreated cells in medium were used as control. Corresponding groups without cells were used as blanks. All experiments were carried out with four replicates.

## 4. Conclusions

In conclusion, our phytochemical investigation of the stems of *S. merrillii* afforded three new triterpenoids: 3*α*,6*α*,30-trihydroxy-ursan-28-oic acid (**1**), 3*α*,30-dihydroxy-6-oxo-ursan-28-oic acid (**2**), 3*α*,6*α*,7*α*,30-tetrahydroxy-ursan-28-oic acid (**3**), together with one known triterpenoid, betulinic acid (**4**) [17], one known anthraquinone, 1,7-dihydroxy-2-methylanthraquinone (**5**) [18], four known phenols, 1,3,5-trimethoxybenzen (**6**) [19], *p*-hydroxybenzoic acid (**7**) [20], syringic acid (**8**) [21], isovanillin (**9**) [22], two steroids, sitosterol (**10**) and daucosterol (**11**). Compound **1** exhibited weak cytotoxicity against the B16F10 cell line with an IC_50_ value of 72.72 μM, while **2** inhibited the proliferation of the A549 cell line with an IC_50_ value of 24.66 μM (Table 2).
