# Correction: Three New Ursane-type Triterpenoids from the Stems of *Saprosma merrillii*. *Molecules* 2013, *18*, 14496-14504

**DOI:** 10.3390/molecules191118618

**Published:** 2014-11-13

**Authors:** Jun-Yan Zhang, Wen-Hao Chen, Da-Shuai Zhang, Guang-Ying Chen, Xue-Ming Zhou, Xiao-Ping Song, Chang-Ri Han

**Affiliations:** Key Laboratory of Tropical Medicinal Plant Chemistry of Ministry of Education, College of Chemistry and Chemical Engineering, Hainan Normal University, Haikou 571158, Hainan, China

The authors wish to inform readers that there are several minor errors and omissions in the chemical structures of compounds **1**–**3** shown in Figure 1 of this paper [1]. Their structures were re-elucidated by detailed analysis of the NMR, MS, and X-ray crystallographic data. The E-ring of **1**–**3** is a five-membered ring, and the hydroxyls of **3** at C-6 and C-7 are both in a *β*-orientation rather than *α*. The corrected Figure 1 is shown below.

**Figure 1:**
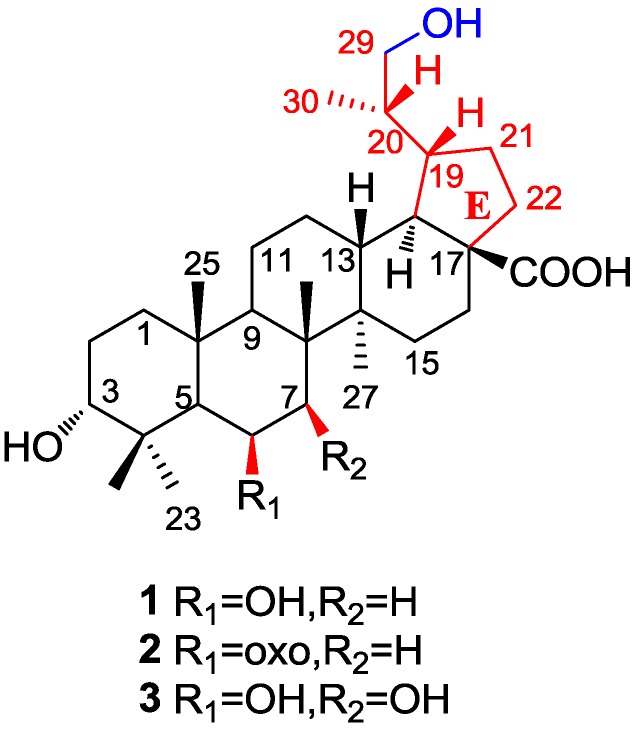
Structures of compounds **1**–**3**.

X-ray Structure Determination of **3** (Figure 2)

Crystal X-ray diffraction data were collected on a Aglient Technologies Gemini A Ultra system with Cu K*α* radiation (λ = 1.54184 Å). The structure was solved by direct methods (SHELXS-97) and refined using full-matrix least-squares difference Fourier techniques. Carbon and oxygen atoms were refined anisotropically. Hydrogen atoms were either refined freely with isotropic displacement parameters or positioned with an idealized geometry and refined riding on their parent C atoms. Crystals suitable for X-ray diffraction **3** was obtained by slow evaporation of a solution in acetone. Crystallographic data (excluding structure factors) for **3** has been deposited with the Cambridge Crystallographic Data Centre: CCDC reference number 1016410. These data can be obtained, free of charge, from the Cambridge Crystallographic Data Centre via http://www.ccdc.cam.ac. uk/data_request /cif. Crystal data for **3**: 3(C_30_H_50_O_6_), C_3_H_6_O_1_, O_1_, 3(H_2_O_1_), M = 1648.23, space group P2_1_, with *α* = 13.1897 (2) Å, *β* = 27.9882(4) Å, *γ* = 13.9241 (3) Å, *α* = 90°, *β* =114.180(2)°, *γ* = 90°, V = 4689.18 (14) Å^3^, Z = 2, T = 293 (2) K, Dc = 1.143 g/mm^3^, *μ* = 0.642 mm^−1^, F (000) =1808, 72328 reflections measured (6.32 ≤ 2*θ* ≤ 134.52), 16709 independent reflections (R_int_ = 0.0369). The final R_1_ values were 0.0490 [*I* > 2*σ*(*I*)]. The final wR*_2_ (F^2^)* values were 0.1399 [*I* > 2*σ*(*I*)]. Flack parameter = 0.00 (12).

**Figure 2:**
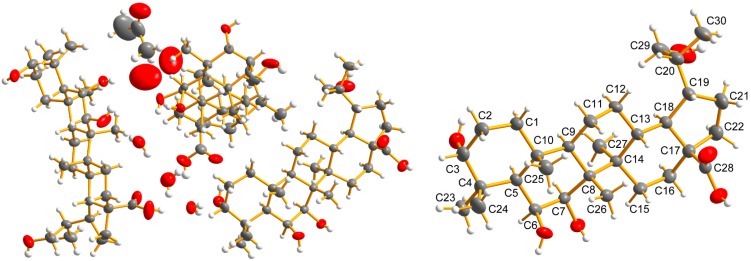
ORTEP drawing of **3**.

Finally, the structures of (3*R*,6*S*,8*R*,10*R*,19*R*,20*R*)-3*α*,6*β*,29-trihydroxylupan-28-oic acid (**1**), (3*R*,8*R*,10*R*,19*R*,20*R*)-3*α*,29-dihydroxy-6-oxo-lupan-28-oic acid (**2**) and (3*R*,6*S*, 7*R*,8*R*,10*R*,19*R*,20*R*) -3*α*,6*β*,7*β*,29-tetrahydroxylupan-28-oic acid (**3**), are revised from the previously reported ursane-type structure to lupane-type triterpenoids.
